# Impact of somatic PI3K pathway and ERBB family mutations on pathological complete response (pCR) in HER2-positive breast cancer patients who received neoadjuvant HER2-targeted therapies

**DOI:** 10.1186/s13058-017-0883-9

**Published:** 2017-07-27

**Authors:** Sinead Toomey, Alexander J. Eustace, Joanna Fay, Katherine M. Sheehan, Aoife Carr, Malgorzata Milewska, Stephen F. Madden, Ausra Teiserskiene, Elaine W. Kay, Norma O’Donovan, William Gallagher, Liam Grogan, Oscar Breathnach, Janice Walshe, Catherine Kelly, Brian Moulton, M. John Kennedy, Guiseppe Gullo, Arnold D. Hill, Colm Power, Deirdre Duke, Niamh Hambly, John Crown, Bryan T. Hennessy

**Affiliations:** 1 Medical Oncology Group, Department of Molecular Medicine, Royal College of Surgeons in Ireland, Dublin 9, Ireland; 2 Department of Histopathology, Royal College of Surgeons in Ireland, Dublin, Ireland; 3 Cancer Trials Ireland, Dublin, Ireland; 4 National Institute for Cellular Biotechnology, Dublin City University, Dublin, Ireland; 5 Cancer Biology and Therapeutics Laboratory, UCD School of Biomolecular and Biomedical Science, UCD Conway Institute,, University College Dublin, Dublin, Ireland; 6 Department of Medical Oncology, Beaumont Hospital, Dublin, Ireland; 7 Department of Medical Oncology, St. Vincent’s University Hospital, Dublin, Ireland; 8 Department of Medical Oncology, The Mater Misericordiae University Hospital, Dublin, Ireland; 9 Department of Medical Oncology, St. James Hospital, Dublin, Ireland; 10 Department of Surgery, Royal College of Surgeons In Ireland, Dublin, Ireland; 11 Department of Radiology, Beaumont Hospital, Dublin, Ireland

**Keywords:** Breast cancer, HER2, Trastuzumab, Lapatinib, PI3K pathway, Somatic mutations

## Abstract

**Background:**

The Cancer Genome Atlas analysis revealed that somatic *EGFR*, receptor tyrosine-protein kinase erbB-2 (*ERBB2*), Erb-B2 receptor tyrosine kinase 3 (*ERBB3*) and Erb-B2 receptor tyrosine kinase 4 (*ERBB4*) gene mutations (ERBB family mutations) occur alone or co-occur with somatic mutations in the gene encoding the phosphatidylinositol 3-kinase (PI3K) catalytic subunit (*PIK3CA*) in 19% of human epidermal growth factor receptor 2 (HER2)-positive breast cancers. Because *ERBB* family mutations can activate the PI3K/AKT pathway and likely have similar canonical signalling effects to PI3K pathway mutations, we investigated their combined impact on response to neoadjuvant HER2-targeted therapies.

**Methods:**

Baseline tumour biopsies were available from 74 patients with HER2-positive breast cancer who were enrolled in the phase II TCHL neoadjuvant study (ICORG 10-05) assessing TCH (docetaxel, carboplatin, trastuzumab) (*n* = 38) versus TCL (docetaxel, carboplatin, lapatinib) (*n* = 10) versus TCHL (docetaxel, carboplatin, trastuzumab, lapatinib) (*n* = 40), each for six cycles. Activating mutations in *PIK3CA* and *ERBB* family genes were identified using mass spectrometry-based genotyping. Phosphatase and tensin homolog (PTEN) expression was assessed by immunohistochemistry.

**Results:**

*PIK3CA* and/or *ERBB* family mutations were detected in 23 (31.1%) tumour samples tested, whereas PTEN expression was low in 31.1% of cases tested. Mutation frequency was similar in each treatment arm (31.3% in TCH arm, 30% in TCL arm and 31.3% in TCHL arm) and was not influenced by oestrogen receptor (ER) status (27.6% in ER-negative patients, 33.3% in ER-positive patients) or progesterone receptor (PR) status (32.6% in PR-negative patients, 29% in PR-positive patients). There was no significant difference in pathological complete response (pCR) rates between 47 patients with wild-type (WT) tumours and 22 patients whose tumours carried mutations (in either *PIK3CA* or *ERBB* family genes) (42.5% vs. 54.5%; *p* = 0.439). Similarly, there was no significant difference in pCR rates between patients with *PIK3CA/ERBB* family mutated/PTEN-low (i.e., PI3K-activated) tumours and patients without PI3K activation (50% vs. 44%; *p* = 0.769). However, in the TCHL (but not the TCH) group, the pCR rate was higher for 9 patients with *PIK3CA/ERBB* family mutated tumours than for 20 patients with *PIK3CA/ERBB* family WT tumours (77.8% vs. 35%; *p* = 0.05).

**Conclusions:**

Our results indicate that patients who receive neoadjuvant TCHL and have *PIK3CA/ERBB* family mutated tumours may be more likely to have a pCR than patients with WT tumours.

**Trial registration:**

ClinicalTrials.gov, NCT01485926. Registered on 2 December 2011.

**Electronic supplementary material:**

The online version of this article (doi:10.1186/s13058-017-0883-9) contains supplementary material, which is available to authorized users.

## Background

Approximately 20% of primary breast cancers have an alteration, usually amplification, of the human epidermal growth factor receptor 2 (*HER2*) gene. *HER2*-amplified cancers have an inferior prognosis, with more frequent and more rapid metastatic relapse [1]. The addition of trastuzumab to conventional chemotherapy has significantly improved the outcomes for patients with both early-stage and metastatic *HER2*-altered breast cancer, with significantly improved survival in both settings [2]. Although a small minority of patients with HER2-positive metastatic disease achieve durable complete remissions, approximately 80% to 90% develop progressive cancer. Addition of trastuzumab to pre-operative chemotherapy results in 30% to 50% of patients achieving a pathological complete response (pCR) [2]. Despite this, approximately 20% of patients with HER2-positive early-stage disease and 90% of patients with metastatic disease will die from disease which is resistant to trastuzumab. Newer targeted anti-HER2 agents have been studied in an attempt to overcome trastuzumab resistance [3–5].

One such agent is lapatinib, a small-molecule inhibitor of epidermal growth factor receptor (EGFR) and receptor tyrosine-protein kinase erbB-2 (ERBB2). In a large randomised trial, the addition of lapatinib to capecitabine in patients whose cancer had progressed following treatment with anthracycline, taxanes and trastuzumab was associated with statistically prolonged progression-free survival compared with capecitabine alone [6]. Evidence suggests that combined targeting of both the extracellular domain of HER2 with trastuzumab and the kinase domain with lapatinib may further improve response. We have shown that combined treatment with trastuzumab and lapatinib improves response to chemotherapy in SKBR3 breast cancer cells and decreases tumour growth in BT-474 xenografts [7], with the combination also showing improved response in the neoadjuvant treatment of HER2-positive breast cancer in some clinical trials [8–11]. However, the benefit of combined HER2-targeted therapy has not been shown consistently. The National Surgical Adjuvant Breast and Bowel Project protocol B-41 demonstrated no significant difference in pCR rates between patients receiving the combination of trastuzumab and lapatinib and those receiving either single agent [12], while the EORTC 10054 study demonstrated a numerically higher but non-significant benefit of double anti-HER2 blockade with trastuzumab and lapatinib [13]. Thus, to determine for ourselves whether the addition of lapatinib to, or the substitution of lapatinib for, trastuzumab would improve pCR, we initiated protocol ICORG 10-05, a prospective, randomised trial with stage Ic/II/III HER2-positive breast cancer patients (NCT01485926) [14].

Activating somatic mutations in the phosphatidylinositol 3-kinase (PI3K)/AKT pathway are present in a range of tumour types [15, 16]. Mutations in *PIK3CA* occur in approximately one-third of breast cancers [17], and these mutations have been implicated in the development of trastuzumab resistance [18, 19]. Ligand binding to ERBB family members activates intracellular signalling pathways such as the PI3K/AKT pathway [20]. Trastuzumab and lapatinib block this signalling, either by binding ERBB2 at the cell surface or by directly inhibiting the kinase activity of both EGFR and ERBB2 [20]. Possible resistance mechanisms include constitutive activation of the PI3K/AKT pathway through somatic mutations in the PI3K pathway or altered intracellular signalling involving loss of phosphatase and tensin homolog (PTEN) [18].

Mutations in *EGFR*, *ERBB2*, Erb-B2 receptor tyrosine kinase 3 (*ERBB3*) and Erb-B2 receptor tyrosine kinase 4 (*ERBB4*) (referred to hereinafter as *ERBB* family mutations) either occur alone or co-occur with *PIK3CA* mutations in 19% of HER2-positive breast cancers (*n* = 58) [21, 22]. Because ERBB-mediated effects are dependent on PI3K/AKT signalling and *ERBB* family mutations can activate the PI3K/AKT pathway, it is likely that they have similar canonical signalling effects to PI3K pathway mutations and PTEN loss. Therefore, the primary aim of our study was to investigate the association of pCR with *ERBB* family and *PIK3CA* mutations and PTEN loss (defined as PI3K pathway activation) in primary HER2-positive breast cancer treated with one or two HER2-targeting agents.

## Methods

### Patient population and samples

TCHL (ICORG10-05) (NCT01485926) is a phase II neoadjuvant study assessing TCH (docetaxel, carboplatin, trastuzumab), TCL (docetaxel, carboplatin, lapatinib) and TCHL (docetaxel, carboplatin, trastuzumab, lapatinib) in stages Ic–III HER2-positive breast cancer patients. Full details of the trial are available at www.clinicaltrials.gov. Eighty-eight patients were randomised to receive either neoadjuvant TCH (six cycles q3weekly (every 3 weeks) docetaxel [75 mg/m^2^] + carboplatin [AUC 6] + trastuzumab 8 mg/kg on day 1 [loading dose] and 6 mg/kg for subsequent cycles, q3weekly (every 3 weeks)), TCL (six cycles q3weekly (every 3 weeks) docetaxel [75 mg/m^2^] + carboplatin [AUC 6] + lapatinib [1000 mg daily until 1 week prior to surgery]) or TCHL (six cycles q3weekly (every 3 weeks) docetaxel [75 mg/m^2^] + carboplatin [AUC 6] + trastuzumab 8 mg/kg on day 1 [loading dose] and 6 mg/kg for subsequent cycles [q3weekly (every 3 weeks)] + lapatinib [1000 mg daily until 1 week prior to surgery]). Patients subsequently underwent surgery and received trastuzumab post-operatively for 1 year from the first dose of trastuzumab. The primary endpoint of the trial was to assess the efficacy of TCH, TCL and TCHL in the neoadjuvant treatment of HER2-positive breast cancer using pCR. Secondary objectives were to assess the clinical response rate and overall response rate in each treatment arm in HER2-positive breast cancer patients and to examine potential molecular and pharmacological markers of response to trastuzumab- and lapatinib-based chemotherapy.

### Sample processing and MassARRAY analysis

Baseline tumour biopsies obtained prior to neoadjuvant chemotherapy were fixed in formalin and embedded in paraffin wax (FFPE). Haematoxylin and eosin staining was performed on 3-μm sections of biopsies and assessed for tumour cellularity by a pathologist. Only samples with greater than 10% tumour cellularity were used for further analysis. DNA extraction was performed using an AllPrep™ DNA/RNA Mini Kit (QIAGEN, Hilden, Germany) as per the manufacturer’s instructions. Mass spectrometry-based single-nucleotide polymorphism genotyping technology (Agena Bioscience, San Diego, CA, USA) was applied to DNA extracted from the FFPE biopsies to detect a total of 108 non-synonymous somatic mutations in *PIK3CA*, *EGFR*, *ERBB2*, *ERBB3* and *ERBB4*. Hot spot mutations in exon 1 (R88Q, K111N), exon 4 (N345K), exon 7 (C420R, E453K), exon 9 (E542V/G/K/Q, E545K/Q/D/A/G/V, Q546H/L/P/R/E/K) and exon 20 (Y1021H/N/C, R1023Q, T1025I/A/S, A1035V/T, M1043V/I, A1046V, H1047R/L/Y, G1049R) of *PIK3CA* were analysed. Mutations in *ERBB* family genes were identified using publicly available data from The Cancer Genome Atlas database and a literature search [22]. AVSIFT and Mutation Assessor scores were used to determine the *ERBB* family mutations that were likely to be deleterious. A full list of mutations is provided in Additional file 1: Table S1. Matrix chips were analysed on a MassARRAY matrix-assisted laser desorption/ionisation time-of-flight mass spectrometry system (Agena Bioscience). Visual inspection and Typer Software were used to identify genotypes on the basis of mass spectra. Reactions where greater than 15% of the resultant mutant mass ran in the mutant site were scored as positive.

### PTEN immunohistochemistry

PTEN immunohistochemistry was performed on 4-μm sections of FFPE tumour biopsies as described previously [23]. Briefly, tissue sections were deparaffinised and rehydrated prior to antigen retrieval at 100 °C for 20 minutes with Bond Epitope Retrieval Solution 1 (Leica Biosystems, Newcastle upon Tyne, UK). Endogenous peroxidase was blocked with 3% peroxidase for 5 minutes. PTEN antibody (clone 6H2.1; Dako/Agilent Technologies, Glostrup, Denmark) was applied at 1:100 dilution, and primary antibody detection was carried out using a polymer system (Bond Polymer Refine Detection; Leica Biosystems). Staining development was achieved by incubation with 3,3′-diaminobenzidine (DAB) and DAB enhancer (Leica Biosystems). PTEN immunohistochemistry was scored by a pathologist as follows. PTEN was considered absent (score = 0) if no immunostaining was detectable in breast carcinoma cells but was present in adjacent benign stromal cells (which acted as an internal control), scored as 1+ if cytoplasmic immunostaining was weak, as 2+ if cytoplasmic immunostaining in carcinoma cells was intermediate, and as 3+ if the cytoplasmic immunostaining in breast carcinoma cells was strong.

### Statistical analysis

Differences in pCR rates by mutation and PTEN status were calculated with a corresponding 95% CI and tested using a χ^2^ test of association. The treatment effect was further examined by performing χ^2^ tests of association on pCR and mutation status and PTEN status for each treatment arm separately.

## Results

### Patient characteristics

Eighty-eight patients were enrolled in the TCHL clinical trial [14]. The median age of the patients at diagnosis was 49 years. Of the 88 patients 53 (60.2%) had oestrogen receptor (ER)-positive tumours, whereas 34 (38.6%) had progesterone receptor (PR)-positive tumours. Thirty-eight (43.2%) of the patients had a pCR (defined as no residual invasive tumour in the breast or lymph nodes at surgery). Also, 38 (43.2%) of the patients received trastuzumab (TCH), whereas 40 (45.4%) of the patients received the combination of lapatinib and trastuzumab (TCHL). Only 10 (11.4%) of the patients received lapatinib alone (TCL), because this arm of the trial was closed early owing to preliminary data emerging from the Adjuvant Lapatinib and/or Trastuzumab Treatment Optimisation (ALTTO) study indicating that TCL treatment was inferior to TCH or TCHL in terms of patient survival outcomes [24]. The full clinical characteristics of the patient cohort are detailed in Table 1.

**Table 1:** Patient clinical characteristics

	TCHL study (*n* = 88)		Sequencing and pCR data available (*n* = 69)		Sequencing, PTEN and pCR data available (*n* = 45)	
Characteristic	No. of patients	%	No. of patients	%	No. of patients	%
ER status						
Negative	35	39.8	28	40.6	19	42.2
Positive	53	60.2	41	59.4	26	57.8
PR status						
Negative	54	61.4	39	56.5	29	64.4
Positive	34	38.6	30	43.5	16	35.6
pCR						
Yes	38	43.2	32	46.4	21	46.7
No	41	46.6	37	53.6	24	53.3
Unknown	9	10.2				
Targeted therapy						
Trastuzumab	38	43.2	30	43.5	21	46.7
Lapatinib	10	11.4	10	14.5	6	13.3
Trastuzumab + lapatinib	40	45.4	29	42	18	40
Age, years						
<49	41	46.6	36	52.2	25	55.6
≥49	47	53.4	33	47.8	20	44.4
Tumour size, cm						
≤5	56	63.6	46	66.7	30	66.7
>5	23	26.1	17	24.6	12	26.6
Unknown	9	10.3	6	8.7	3	6.7
N stage						
N0	25	28.4	20	29	9	20
N1	50	56.8	40	58	31	68.9
N2	2	2.3	1	1.4	1	2.2
NX	2	2.3	2	2.9	1	2.2
Unknown	9	10.2	6	8.7	3	6.7
M stage						
M0	88	100	69	100	45	100
Overall stage						
IIA	30	34.1	26	37.7	12	26.6
IIB	31	35.2	25	36.2	21	46.7
IIIA	7	8	4	5.8	3	6.7
IIIB	10	11.4	8	11.6	6	13.3
IIIC	1	1.1	0	0	0	0
Unknown	9	10.2	6	8.7	3	6.7

*Abbreviations: ER* Oestrogen receptor, *PR* Progesterone receptor, *pCR*, Pathological complete response; *PTEN* Phosphatase and tensin homolog, *TCHL* Docetaxel, carboplatin, trastuzumab, lapatinib

A total of 88 patients were enrolled in the TCHL phase II neoadjuvant study. After quality control, 74 patient samples were genotyped. Of these 74 samples, pCR data were available for 69 patients. Forty-five tumour samples had sufficient material for PTEN analysis. The clinical characteristics of these three patient groups are shown

### Somatic mutation profiling

**Fig. 1:**
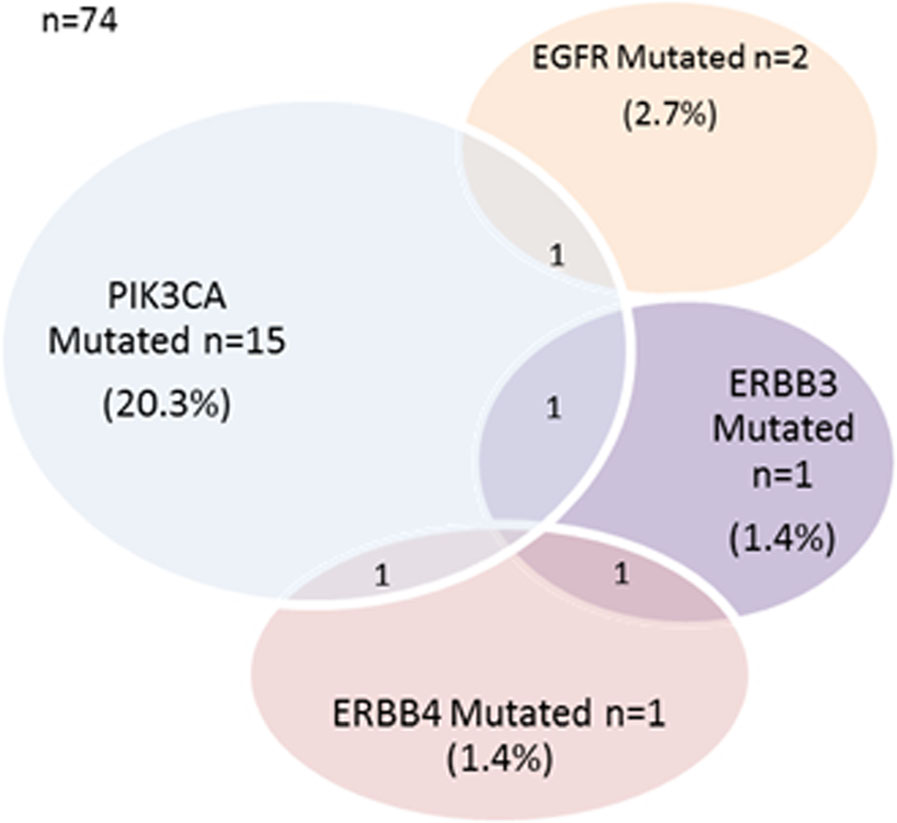
Frequency of somatic *PIK3CA* and/or *ERBB* family mutations in baseline tumour biopsies from 74 patients enrolled in the TCHL clinical trial where adequate tumour was available following quality control. *EGFR* Epidermal growth factor receptor, *ERBB3* Erb-B2 receptor tyrosine kinase 3, *ERBB4* Erb-B2 receptor tyrosine kinase 4

Each biopsy taken prior to neoadjuvant chemotherapy was assessed for tumour cellularity by a pathologist, and samples with less than 10% tumour material or that produced low DNA yield were excluded from the analysis, which left a total of 74 tumour samples for sequencing analysis. MassARRAY analysis was used to identify hot spot mutations in *PIK3CA*, *EGFR*, *ERBB2*, *ERBB3* and *ERBB4. PIK3CA* mutations alone were detected in 15 breast tumour samples (20.3%), whereas *EGFR* mutations alone were detected in 2 samples (2.7%). *ERBB3* and *ERBB4* mutations alone were each detected in one sample (1.4%). No activating mutations were detected in *ERBB2. EGFR*, *ERBB3* and *ERBB4* mutations co-occurred with *PIK3CA* mutations in 1 sample each (1.4%), whereas 1 sample had co-occurring *ERBB3* and *ERBB4* mutations (1.4%) (Fig. 1), giving an overall *PIK3CA* and/or *ERBB* mutation frequency of 31.1% (23 samples). This overall mutation frequency was similar in each treatment arm (30% in TCL arm [three samples], 31.3% in TCH arm [ten samples], and 31.3% in TCHL arm [ten samples]) and was not influenced by ER status (27.6% in ER-negative tumours, 33.3% in ER-positive tumours) or PR status (32.6% in PR-negative tumours, 29% in PR-positive tumours) (Table 2). Specific mutations identified are detailed in Additional file 2.

**Table 2:** Somatic mutation genotyping results by oestrogen receptor and progesterone receptor status and treatment arm

		PIK3CA mutated	ERBB mutated	PIK3CA/ERBB mutated^a^
Tumour group	Total no. of tumours	No. of tumours (%)	No. of tumours (%)	No. of tumours (%)
ER status				
Negative	29	8 (27.6)	1 (3.4)	8 (27.6)
Positive	45	10 (22.2)	7 (15.6)	15 (33.3)
PR status				
Negative	43	9 (20.9)	5 (11.6)	14 (32.6)
Positive	31	8 (25.8)	3 (9.7)	9 (29)
Treatment arm				
TCL	10	3 (30)	2 (20)	3 (30)
TCH	32	9 (28.1)	2 (6.3)	10 (31.3)
TCHL	32	6 (18.8)	4 (12.5)	10 (31.3)

*Abbreviations: ER* Oestrogen receptor, *PR* Progesterone receptor, *TCH* Docetaxel, carboplatin, and trastuzumab, *TCHL* Docetaxel, carboplatin, trastuzumab, lapatinib, *TCL* Docetaxel, carboplatin, lapatinib

Association of *PIK3CA* mutations, *ERBB* family mutations and combined *PIK3CA/ERBB* family mutations with ER and PR status. The mutation frequency in each treatment arm is also shown

^a^These numbers/percentages may not be a sum of *PIK3CA* and *ERBB* mutated because of mutation overlap

### PTEN expression and its overlap with mutation status

**Fig. 2:**
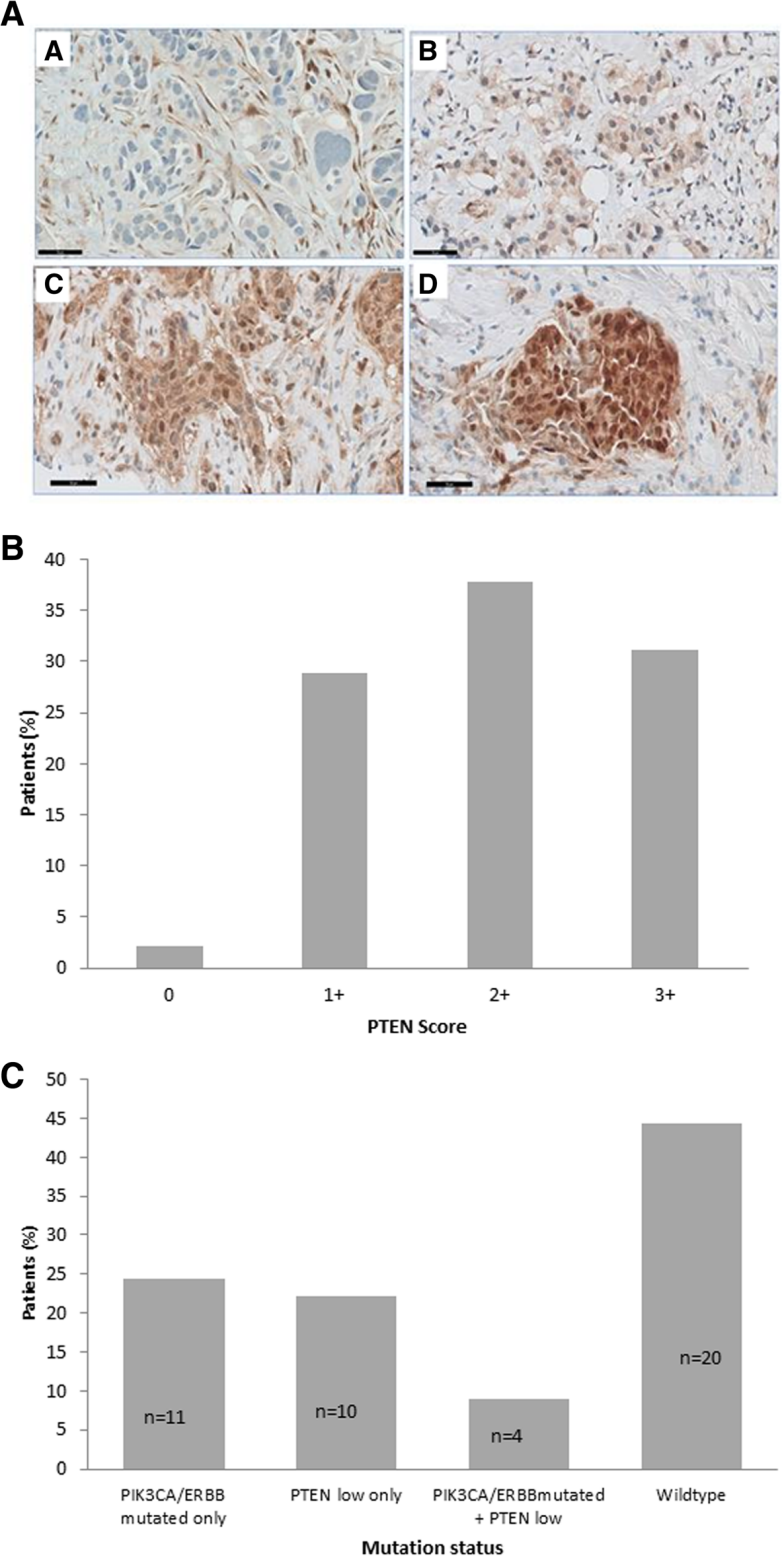
**a** Immunohistochemical staining patterns of phosphatase and tensin homolog (PTEN). No immunostaining was detectable in breast carcinoma cells, but it was present in adjacent benign stromal cells (0; *A*), cytoplasmic immunostaining weak (1+; *B*), cytoplasmic immunostaining in carcinoma cells intermediate (2+; *C*), cytoplasmic immunostaining strong (3+; *D*) (scale bars = 50 μm). **b** Frequency of tumours with 0, 1+, 2+ and 3+ immunostaining. All images were taken at × 40 original magnification. **c** Frequency of *PIK3CA/ERBB* mutations, low expression of PTEN, and co-occurring *PIK3CA/ERBB* mutations and low expression of PTEN (i.e., PI3K pathway activated) in 45 baseline tumour biopsies that were evaluable for PTEN expression

We examined the expression of PTEN protein by immunohistochemical analysis in the diagnostic biopsies of 45 tumours where there was sufficient tissue for PTEN evaluation. Representative staining patterns for each parameter are shown in Fig. 2a. Fourteen (31.1%) of 45 tumours were considered to have low (absent or weak) PTEN expression (0/1+) in the tumour (Fig. 2b). No differences in expression rates were seen among the three different treatment arms (Table 3). Low PTEN expression, although less frequent in cases with concurrent *PIK3CA/ERBB* mutations (10 [22.2%] of 45 vs. 4 [8.9%] of 45), was not mutually exclusive with *PIK3CA/ERBB* mutations (Fig. 2c). Low PTEN expression was more frequently seen in PR-negative than in PR-positive tumours (37.9% vs. 18.8%); however, there was no difference in the frequency of low PTEN expression between ER-negative and ER-positive tumours (31.6% vs. 30.8%) (Table 3). PI3K pathway activation (defined by a *PIK3CA/ERBB* mutation and/or low expression of PTEN) was found in 25 (55.6%) tumours overall and was not influenced by either ER or PR status: 57.9% of ER-negative tumours and 53.8% of ER-positive tumours had an activated PI3K pathway, whereas 58.6% of PR-negative and 50% of PR-positive tumours had an activated PI3K pathway. PI3K pathway activation also occurred at similar frequencies in each treatment arm (66.7% in TCL arm [4 samples], 61.9% in TCH arm [13 samples] and 44.4% in TCHL arm [8 samples]) (Table 3).

**Table 3:** Somatic mutation genotyping and phosphatase and tensin homolog immunohistochemistry results by oestrogen receptor and progesterone receptor status and treatment arm

		PTEN low	PIK3CA/ERBB mutated and/or PTEN low^a^
Patient group	Total no. of patients	No. of patients (%)	No. of patients (%)
ER status			
Negative	19	6 (31.6)	11 (57.9)
Positive	26	8 (30.8)	14 (53.8)
PR status			
Negative	29	11 (37.9)	17 (58.6)
Positive	16	3 (18.8)	8 (50)
Treatment arm			
TCL	6	2 (33.3)	4 (66.7)
TCH	21	7 (33.3)	13 (61.9)
TCHL	18	5 (31.3)	8 (44.4)

*Abbreviations: ER* Oestrogen receptor, *PR* Progesterone receptor, *TCH* Docetaxel, carboplatin, and trastuzumab, *TCHL* Docetaxel, carboplatin, trastuzumab, lapatinib, *TCL* Docetaxel, carboplatin, lapatinib

Association of low PTEN expression and combined low PTEN expression and PIK3CA/ERBB family mutations (i.e., PI3K pathway activation) with ER and PR status. The mutation frequency in each treatment arm is also shown

^a^Because of mutation overlap, these numbers/percentages may not be a sum of PIK3CA and ERBB mutated

### Mutation and PTEN status and pCR

**Fig. 3:**
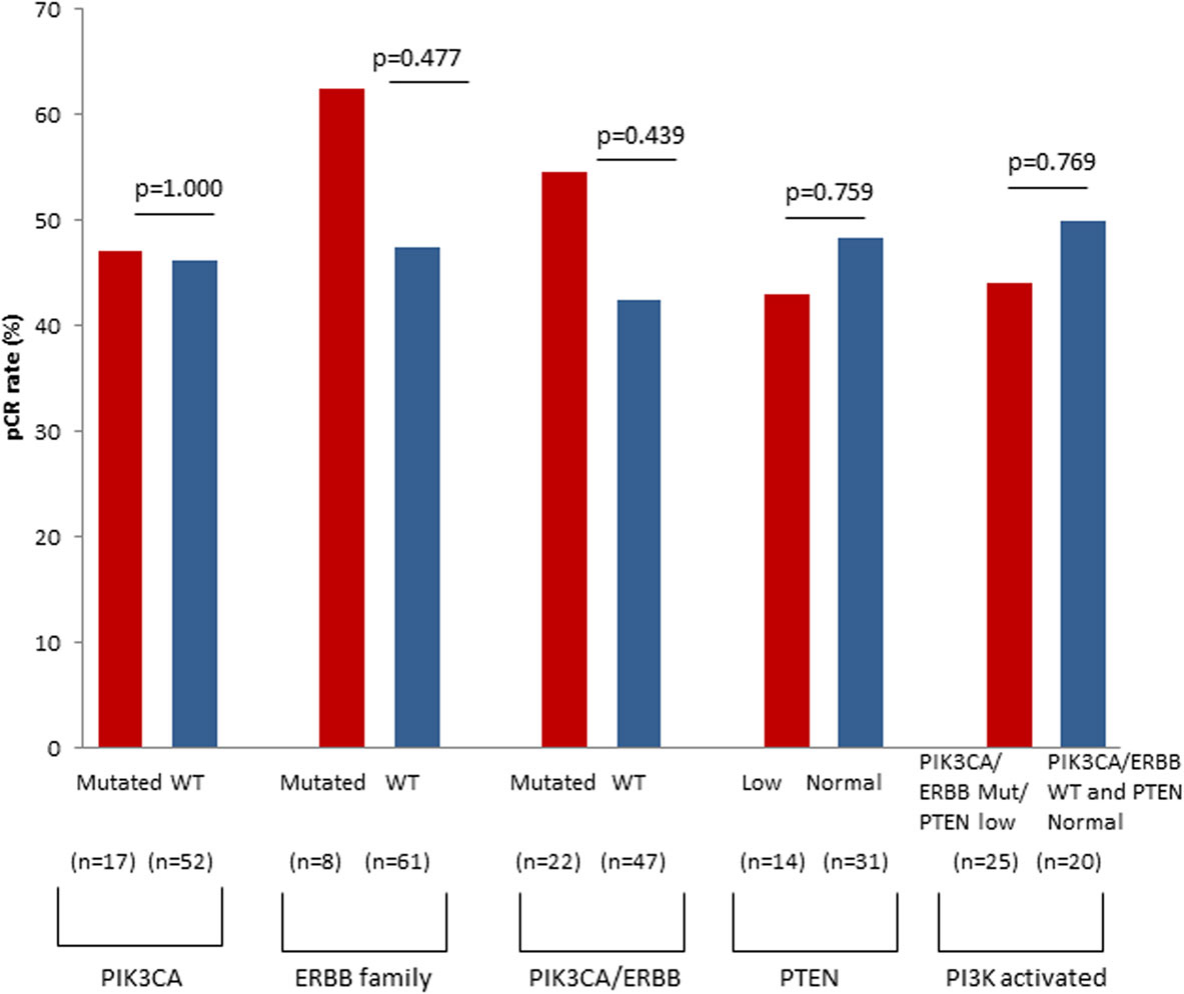
Influence of *PIK3CA* and *ERBB* mutations and phosphatase and tensin homolog (PTEN) expression status on pathological complete response (pCR) rates using a χ^2^ test of association. The proportion of patients who obtained pCR is shown for the entire patient cohort. Phosphatidylinositol 3-kinase (PI3K) activation is defined as the presence of one or more of the following in a breast tumour: *PIK3CA* or *ERBB* family mutations or low PTEN expression. *WT* Wild type

In the combined TCH/TCHL study population, we analysed the association between tumour mutation status and pCR, as defined by no invasive cancer in the breast and no involvement of axillary nodes in 69 patients where pCR data were available (Fig. 3). There was no significant difference in pCR rates between 17 patients with *PIK3CA* mutated vs. 52 patients with *PIK3CA* wild-type (WT) tumours (47% vs. 46.1%; *p* = 1.000) or between 8 patients with *ERBB* family mutated vs. 61 patients with *ERBB* family WT tumours (62.5% vs. 47.5%; *p* = 0.477). When we combined tumours with *PIK3CA* and *ERBB* family mutations, there was no difference in pCR rates between 22 patients with mutated vs. 47 patients with WT tumours (54.5% vs. 42.6%; *p* = 0.439) (Fig. 3). We also found no difference in pCR rates between 14 patients with low tumour PTEN expression and 31 patients with moderate or strong PTEN expression in their tumours (42.9% vs. 48.4%; *p* = 0.759). When we combined tumours with a *PIK3CA/ERBB* family mutation and/or PTEN loss (i.e., PI3K pathway activated), we found no difference in pCR rates between 25 patients with PI3K activated tumours and 20 patients with tumours without an activated PI3K pathway (44% vs. 50%; *p* = 0.769) (Fig. 3).

**Fig. 4:**
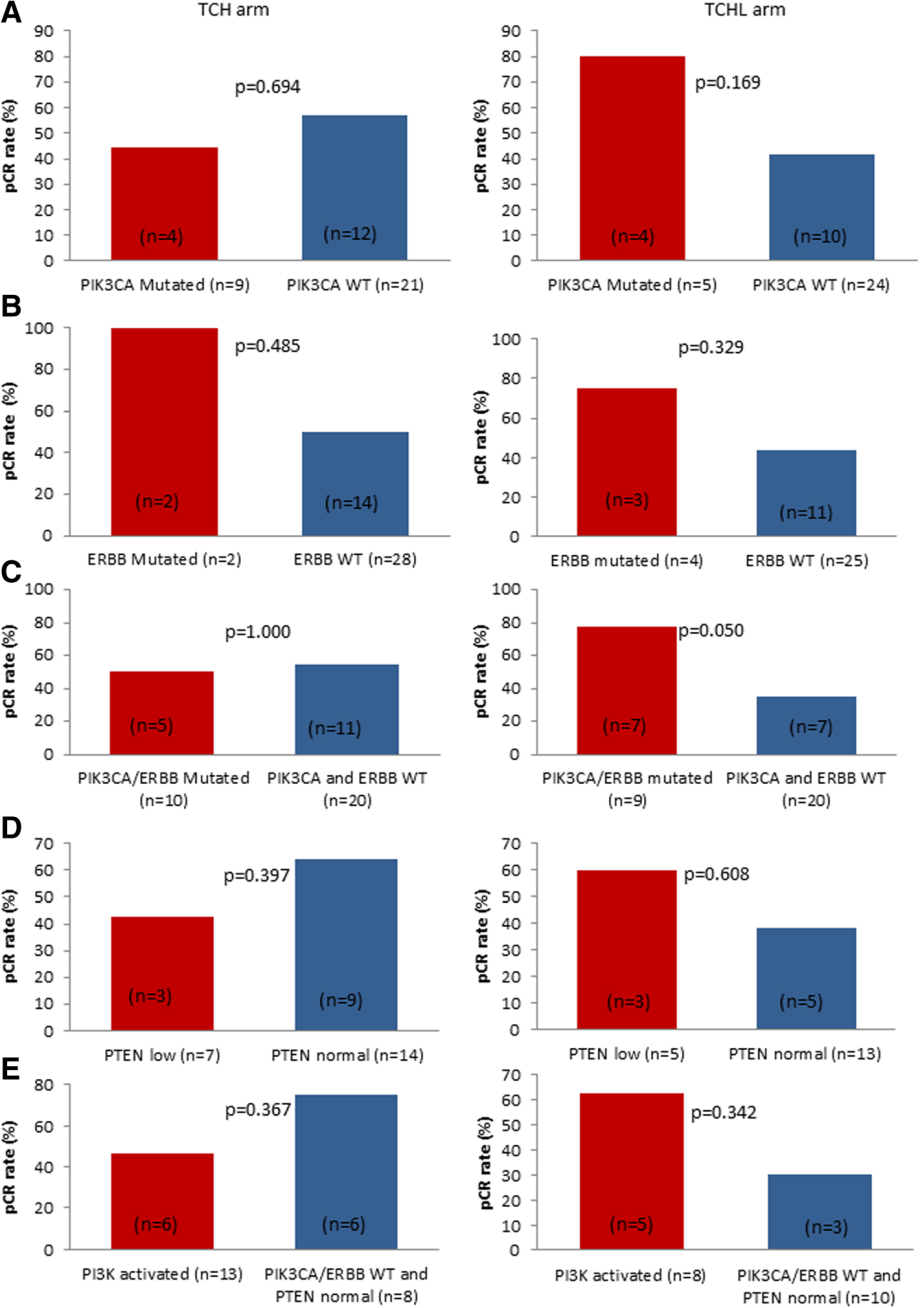
Influence of mutation and phosphatase and tensin homolog (PTEN) status on pathological complete response (pCR) rate by treatment arm using a χ^2^ test of association. **a** Influence of *PIK3CA* mutations in tumours on pCR in patients receiving TCH (docetaxel, carboplatin, trastuzumab) and TCHL (docetaxel, carboplatin, trastuzumab, lapatinib). **b** Influence of *ERBB* family mutations in tumours on pCR in patients receiving TCH and TCHL. **c** Influence of combined *PIK3CA* and *ERBB* family mutation status in tumours on pCR in patients receiving TCH and TCHL. **d** Influence of low PTEN expression in breast tumours on pCR in patients receiving TCH and TCHL. **e** Influence of tumour phosphatidylinositol 3-kinase (PI3K) activation status on pCR in patients receiving TCH and TCHL. PI3K activation is defined as the presence of one or more of the following in a breast tumour: *PIK3CA* or *ERBB* family mutations or low PTEN expression. *WT* Wild type

There was no difference in overall pCR rates in each treatment arm (Table 4); however, we also specifically examined the influence of mutation status on pCR in the TCH and TCHL arms (Fig. 4). There was no difference in pCR rates in patients with either *PIK3CA*-mutated tumours or *ERBB* family-mutated tumours compared with patients with WT tumours in either the TCH or the TCHL arm (Fig. 4a and b); however, when we combined *PIK3CA* and *ERBB* family mutations, in the TCHL arm, the pCR rate was higher for 9 patients whose tumours harboured a *PIK3CA* and/or an *ERBB* family mutation compared with 20 patients with WT tumours (77.8% vs. 35%; *p* = 0.05) (Fig. 4c). There was no difference in pCR rates between these two groups of breast tumours in the TCH arm. The results shown in Fig. 4c suggest that a trend may exist in the data towards a benefit of TCHL treatment in patients with *PIK3CA* and/or *ERBB* family mutations, and although this was not significant after multiple testing (unadjusted *p* value = 0.05), it warrants further study in a larger cohort. When we included patients with low tumour expression of PTEN, there was no difference in pCR rates in patients whose tumours had a *PIK3CA/ERBB* family mutation and/or low PTEN expression (i.e., PI3K activated) compared with patients with WT tumours in either the TCH or the TCHL arm (Fig. 4d and e).

**Table 4:** Overall pathological complete response rates in the three treatment arms

Response	TCL (*n* = 10)	TCH (*n* = 36)	TCHL (*n* = 33)
pCR (no invasive cancer in the breast or LNs)	2 (20%)	19 (52.8%)	17 (51.5%)
*p* = 0.0839 (TCL vs. TCH); 1.000 (TCH vs. TCHL); 0.1488 (TCL vs. TCHL)			

*Abbreviations: LN* Lymph node, *pCR* Pathological complete response, *TCH* Docetaxel, carboplatin, and trastuzumab, *TCHL* Docetaxel, carboplatin, trastuzumab, lapatinib, *TCL* Docetaxel, carboplatin, lapatinib

Overall pCR rates within each treatment arm following 6 cycles of neoadjuvant TCL, TCH or TCHL

Figure 4c shows that 9 of 29 tumours had *PIK3CA* or *ERBB* mutations. Figure 4d shows that 5 of 18 tumours had PTEN deficiency. Figure 4e shows that 8 of 18 tumours had mutations or PTEN deficiency, meaning that only 3 of the *PIK3CA/ERBB*-mutant tumours (from Fig. 4c) are included in the composite analysis in Fig. 4e.

## Discussion

Biomarker analysis is necessary to identify those patients most likely to respond to neoadjuvant chemotherapy. In HER2-positive breast cancer, HER2 status is still the only recognised predictive marker to select patients for anti-HER2-targeted therapy; however, in recent years, a number of other biomarkers have emerged as potential predictors for response. PI3K pathway activation is the most common signal transduction pathway alteration in breast cancer [17, 25]. It mostly results from somatic *PIK3CA* mutations or PTEN loss [26], but it can also be a result of mutations in *ERBB* family genes [20, 27]. Although a number of studies have reported on PI3K pathway activation in HER2-positive breast cancer, they have been focused mainly on its impact on response to anti-HER2 therapy in metastatic disease or in the adjuvant setting [18, 28–30]. In the present study, we sought to evaluate associations between activation of the PI3K pathway and the efficacy of trastuzumab and lapatinib therapy in the neoadjuvant setting in early HER2-positive breast cancer.

Whereas pre-clinical studies have shown that PI3K pathway activation contributes to resistance to anti-HER2-targeted therapies [18, 31], clinical studies have failed to give a clear answer. In the Neoadjuvant Lapatinib and/or Trastuzumab Treatment Optimization Trial (NeoALTTO), tumours with *PIK3CA* mutations had lower pCR rates after treatment with neoadjuvant paclitaxel plus HER2-targeted therapy, with pCR rates decreasing from 34.5% in *PIK3CA* WT tumours to 21.3% in mutated tumours [32]. Results from two sequential neoadjuvant studies (NCT00133796 and NCT00206427) demonstrated that patients with tumours with low PTEN expression or PI3K pathway mutations were less likely to achieve a pCR after neoadjuvant trastuzumab/docetaxel than patients with tumours with no PI3K pathway mutations or high PTEN expression (18.2% vs. 66.7%; *p* = 0.015) [33]. In the Chemotherapy, Herceptin and Lapatinib in Operable Breast cancer (CHER-LOB) study, similar pCR rates after neoadjuvant paclitaxel for 12 weeks followed by fluorouracil, epirubicin and cyclophosphamide for four courses every 3 weeks plus HER2-targeted therapy were seen in patients with *PIK3CA*-WT and *PIK3CA*-mutated breast cancers (33.3% vs. 22.7%; *p* = 0.323) [34]. A recent meta-analysis of pooled data from five neoadjuvant clinical trials demonstrated lower pCR rates in the *PIK3CA* mutant cohort than in the WT cohort (16.2% vs. 29.6%; *p* < 0.001) [35].

In the present study, somatic *PIK3CA* mutations were found in 24.3% of tumours, and *ERBB* family mutations were found in 10.8% of tumours, with a similar distribution in ER-positive or ER-negative tumours, which confirms results of previous studies [17, 21, 22]. Reduced PTEN expression was found in 31.1% of tumours. Combining these aberrations which result in activation of the PI3K pathway, we find 55.6% of tumours with PI3K pathway activation (Fig. 2c). However, there was no correlation between activation of the PI3K pathway and response to anti-HER2 neoadjuvant therapy in all study patients as measured by pCR (Fig. 3). The rate of pCR was similar among tumours with activation of the PI3K pathway (defined as a *PIK3CA* mutation and/or low PTEN expression and/or an *ERBB* family mutation) and among tumours without a *PIK3CA* or *ERBB* family mutation or low PTEN expression (44% vs. 50%; *p* = 0.769) (Fig. 3).

Pre-clinical studies suggest that PTEN loss may be a potential mechanism of resistance to HER2-targeted therapies [36, 37]. In the neoadjuvant setting, results are more variable. Dave et al. [33] showed that patients with low PTEN expression who received trastuzumab had lower pCR rates than those with high PTEN expression (15.4% vs. 44.4%). However, in those patients who received lapatinib, low expression of PTEN was significantly associated with higher pCR rates than high expression (92.3% vs. 41.2%; *p* = 0.007). In the GeparQuattro study, low PTEN expression was also associated with lower response to trastuzumab-based chemotherapy compared with high PTEN expression (27.6% vs. 57.1%; *p* = 0.010) [38]. In our present study, we did not find any association between PTEN expression and pCR, similar to the results of the NeoALTTO study [39]. The differences between the studies may be attributable to a lack of standardisation of PTEN detection and scoring methods, as well as to the lack of a standardised definition for low and high PTEN expression.

In the TCH arm, as in all study patients, the pCR rates were not significantly affected by *PIK3CA* or *ERBB* family mutations or by PTEN or PI3K activation status. In contrast, in the TCHL arm, patients with *PIK3CA* and/or *ERBB* family mutated tumours were more likely to achieve a pCR than patients with *PIK3CA* and *ERBB* WT tumours (77.8% vs. 35%; *p* = 0.05) (Fig. 3). Our results are in contrast to NeoALTTO, where patients treated with a combination of weekly paclitaxel, trastuzumab and lapatinib who had *PIK3CA*-WT tumours had a pCR rate of 53.1%, which decreased to 28.6% in patients with tumours harbouring *PIK3CA* mutations [32]. The difference in the impact of *PIK3CA* mutations in the two studies may be attributable to the low sample size in the TCHL cohort. Our study also included analysis of additional biomarkers of PI3K/AKT activation, including *ERBB* family mutations, which suggests that a trend may exist within the data towards a benefit of TCHL treatment in patients with *PIK3CA* and/or *ERBB* family mutations, and this warrants further study in a larger cohort. Furthermore, it is worth noting that the neoadjuvant regimens used are different in the relevant study arms. TCHL for six cycles was used in our study versus HL (trastuzumab and lapatinib) followed by weekly paclitaxel for 12 weeks with HL in NeoALLTO; therefore, sensitivity to the chemotherapy part of the regimen itself cannot be ruled out.

The finding that activating mutations in a signalling pathway can increase sensitivity to targeted therapies is not unusual. The tyrosine kinase inhibitors erlotinib and gefitinib show significant clinical responses in lung adenocarcinoma patients harbouring *EGFR* activating mutations [40], whereas dacomitinib, a pan-HER tyrosine kinase inhibitor has been shown to be beneficial in *HER2*-mutated lung tumours [41]. *ERBB4* has been shown to be highly mutated in melanoma, where *ERBB4* mutations increase ERBB4 kinase activity despite similar expression of the HER4 protein and sensitise cells to lapatinib [42]. In some, though not all, clinical studies, the combination of trastuzumab and lapatinib has been shown to be more effective than each drug given alone, likely because acquired drug resistance that results from the activation of alternate pro-survival pathways can be overcome by combination treatments [43]. In vitro, while *PIK3CA* mutated HER2-positive BT474 cells stably expressing the *ERBB2*-T798M mutation were resistant to trastuzumab, the addition of lapatinib or cetuximab restored sensitivity [44], likely through blocking heterodimer formation [44].

A limitation of our study and other studies is the absence of a group not treated with anti-HER2 therapy. Given that all patients received chemotherapy in association with anti-HER2 treatment, and also that there was considerable heterogeneity in the chemotherapy treatment regimens used between the studies, we cannot exclude the possibility that some of our findings are contributed to by sensitivity to the chemotherapy part of the regimen itself. We also acknowledge that our study is small, and it may be useful to validate these results in a larger cohort, where there will be a greater power to demonstrate a positive interaction between mutation status and pCR.

## Conclusions

To the best of our knowledge this is the first study looking at the effects of PI3K pathway activation through a combination of *PIK3CA* gene mutations, *ERBB* family gene mutations and low PTEN expression on pCR in the neoadjuvant treatment of HER2-positive breast cancer. Our study confirms the high prevalence of mutations that result in activation of the PI3K pathway in HER2-positive breast cancer and their potential utility in predicting patients most likely to respond to combination anti-HER2 therapy. Because this is also the first report on the impact of *PIK3CA* mutations on responsiveness to neoadjuvant taxane plus carboplatin-based HER2-targeted therapy regimens, with other studies such as NeoALLTO involving neoadjuvant taxane- or taxane/anthracycline-based HER2-targeted therapy regimens, our findings need validation in other studies.

## Additional files

Additional file 1: Table S1. Non-synonymous somatic mutations in *PIK3CA* and *ERBB* family genes. (DOCX 15 kb)

Additional file 2: Table S2. Specific somatic mutations in *PIK3CA*, *EGFR*, *ERBB3*, *ERBB4* and *PIK3CA* detected in our patient cohort. (DOCX 13 kb)

## Abbreviations

ALTTO: Adjuvant Lapatinib and/or Trastuzumab Treatment Optimisation
CHER-LOB: Chemotherapy, Herceptin and Lapatinib in Operable Breast cancer study
DAB: 3,3′-Diaminobenzidine
EGFR: Epidermal growth factor receptor
ER: Oestrogen receptor
ERBB2: Receptor tyrosine-protein kinase erbB-2
ERBB3: Erb-B2 receptor tyrosine kinase 3
ERBB4: Erb-B2 receptor tyrosine kinase 4
FFPE: Fixed in formalin and embedded in paraffin wax
HER2: Human epidermal growth factor receptor 2
LN: Lymph node
NeoALTTO: Neoadjuvant Lapatinib and/or Trastuzumab Treatment Optimization Trial
pCR: Pathological complete response
PI3K: Phosphatidylinositol 3-kinase
PR: Progesterone receptor
PTEN: Phosphatase and tensin homolog
TCH: Docetaxel, carboplatin, trastuzumab
TCHL: Docetaxel, carboplatin, trastuzumab, lapatinib
TCL: Docetaxel, carboplatin, lapatinib
WT: Wild type
