# Supplementary material for: Impact of somatic PI3K pathway and ERBB family mutations on pathological complete response (pCR) in HER2-positive breast cancer patients who received neoadjuvant HER2-targeted therapies
Source: Breast Cancer Res. 2017 Jul 27;19:87. doi: 10.1186/s13058-017-0883-9 (PMC5530949; doi:10.1186/s13058-017-0883-9)
Supplement: Supplementary file 1 — Non-synonymous somatic mutations in PIK3CA and ERBB family genes. (DOCX 15 kb) [file 13058_2017_883_MOESM1_ESM.docx]

| **EGFR** | **ERBB2** | **ERBB3** | **ERBB4** | **PIK3CA** |
| --- | --- | --- | --- | --- |
| G179A/D | S310F/Y | V104M | S303Y/F | R88Q |
| V689M | L755S | A232V | V348M/L | K111N |
| N700D | D769H | P262H | D595G/V | N345K |
| E709A/V/G/K/Q | G776S/V | G284R | V721I | C420R |
| G719C/S/R | V777L/M/A | D297Y | R782Q | E453K |
| S720T/P | V842I | T355A | E810K | E542V/G/K/Q |
| D761N/Y | H878Y | G325R | P854Q | E545K/Q/D/A/G/V |
| V769L/M |  | T389K | N861Y | Q546H/L/P/R/E/K |
| T783A |  | V714M | E872K/V | Y1021H/N/C |
| A839T |  | Q809R | E874X | R1023Q |
| K846R |  | S846I | T926M | T1025I/A/S |
| L858M |  | E928G | E934K | A1035V/T |
| L858R |  |  | K935T/R/I | M1043V/I |
| L861Q/R  G863D  H870R |  |  | K935E  G936R | A1046V  H1047R/L/Y  G1049R |
|  |  |  |  |  |

Table S1: Nonsynonymous somatic mutations in PIK3CA and ERBB family genes

108 Nonsynonymous mutations in EGFR, ERBB2, ERBB3, ERBB4 and PIK3CA analysed by Agena MassArray in this study
