# Supplementary material for: Impact of somatic PI3K pathway and ERBB family mutations on pathological complete response (pCR) in HER2-positive breast cancer patients who received neoadjuvant HER2-targeted therapies
Source: Breast Cancer Res. 2017 Jul 27;19:87. doi: 10.1186/s13058-017-0883-9 (PMC5530949; doi:10.1186/s13058-017-0883-9)
Supplement: Supplementary file 2 — Specific somatic mutations in PIK3CA, EGFR, ERBB3, ERBB4 and PIK3CA detected in our patient cohort. (DOCX 13 kb) [file 13058_2017_883_MOESM2_ESM.docx]

| Patient | Arm | PIK3CA | EGFR | ERBB3 | ERBB4 |
| --- | --- | --- | --- | --- | --- |
| 3 | TCL | H1047R | WT | WT | WT |
| 5 | TCHL | H1047R | WT | WT | WT |
| 19 | TCL | H1047R | WT | WT | S303F |
| 20 | TCHL | H1047R | WT | WT | WT |
| 21 | TCHL | WT | K846R | WT | WT |
| 25 | TCL | E542K | V398I | WT | WT |
| 28 | TCH | H1047R | WT | WT | WT |
| 30 | TCHL | WT | WT | WT | S303F |
| 32 | TCH | H1047R | WT | WT | WT |
| 33 | TCH | E545K | WT | WT | WT |
| 44 | TCHL | E545K | WT | WT | WT |
| 46 | TCH | H1047R | WT | WT | WT |
| 49 | TCHL | H1047R | WT | WT | WT |
| 51 | TCHL | WT | WT | Q809R | S303F |
| 52 | TCH | WT | WT | T355I | WT |
| 59 | TCH | H1047R | WT | WT | WT |
| 62 | TCHL | H1047R | WT | WT | WT |
| 68 | TCH | H1047R | WT | WT | WT |
| 71 | TCHL | H1047R | WT | WT | WT |
| 74 | TCH | H1047R | WT | WT | WT |
| 76 | TCH | H1047R | WT | WT | WT |
| 80 | TCHL | WT | V769L | WT | WT |
| 83 | TCH | H1047R | WT | Q809R | WT |

Table S2. Specific somatic mutations in PIK3CA, EGFR, ERBB3, ERBB4 and PIK3CA detected in our patient cohort
